# Expanding the role of PSMA PET in active surveillance

**DOI:** 10.1186/s12894-023-01219-4

**Published:** 2023-04-29

**Authors:** Anika Jain, Anthony-Joe Nassour, Thomas Dean, Imogen Patterson, Lisa Tarlinton, Lawrence Kim, Henry Woo

**Affiliations:** 1 Department of Urology, Sydney Adventist Hospital, Sydney, Australia; 2 SAN Prostate Centre of Excellence, Sydney Adventist Hospital, Sydney, Australia; 3 College of Health and Medicine, Australian National University, Sydney, Australia

**Keywords:** Prostate cancer, Active surveillance, PSMA PET

## Abstract

**Introduction:**

Accurate grading at the time of diagnosis is fundamental to risk stratification and treatment decision making, particularly for men being considered for Active Surveillance (AS). With the introduction of prostate-specific membrane antigen (PSMA) positron emission tomography (PET) there has been considerable improvement in sensitivity and specificity for the detection and staging of clinically significant prostate cancer. Our study aims to determine the role of PSMA PET/CT in men with newly diagnosed low or favourable intermediate risk prostate cancer to better select men for AS.

**Method:**

This is a retrospective single centre study performed from January 2019 and October 2022. This study includes men identified from electronic medical record system who had undergone a PSMA PET/CT following newly diagnosed low or favourable-intermediate risk prostate cancer. Primary outcome was to assess the change in management for men being considered for AS following PSMA PET/CT results on the basis of PSMA PET characteristics.

**Results:**

In total, there were 11 of 30 men (36.67%) who were assigned management by AS and 19 of 30 men (63.33%) who had definitive treatment. 15 of the 19 men that needed treatment had concerning features on PSMA PET/CT results. Of the 15 men with concerning features on PSMA PET, 9 (60%) men were found to have adverse pathological features on final prostatectomy features.

**Conclusion:**

This retrospective study suggests that PSMA PET/CT has potential to influence the management of men with newly diagnosed prostate cancer that would otherwise be appropriate for active surveillance.

**Supplementary Information:**

The online version contains supplementary material available at 10.1186/s12894-023-01219-4.

## Introduction

Prostate cancer is the second most common cause of cancer death after lung cancer [1]. A variety of treatment options are now available for men with localised disease. Active surveillance (AS) has become a widely used strategy for men with low-risk prostate cancer. It aims to avoid overtreatment, but at the same time carries an acceptable risk associated with treatment delay should their cancer necessitate intervention [2]. Patients remain under close surveillance through structured programmes with regular follow-up consisting of PSA testing, clinical examination, multi-parametric magnetic resonance imaging (mpMRI) imaging and repeat prostate biopsies, with curative treatment being prompted by pre-defined thresholds.

Accurate grading at the time of diagnosis is fundamental to risk stratification and treatment decision making, particularly for men being considered for AS. With the introduction of prostate-specific membrane antigen (PSMA) positron emission tomography (PET) there has been considerable improvement in sensitivity and specificity for the detection and staging of primary or recurrent prostate cancer [3–5]. Several studies have demonstrated that tumour uptake, represented by PSMA expression, is strongly correlated with Gleason Score of the primary prostatic tumour [6, 7]. This emerging imaging modality is a useful diagnostic tool to help identify men with clinically significant prostate cancer and therefore has the potential to improve patient selection of men suitable for active surveillance.

Multiple studies have investigated cohorts of men on active surveillance, with good long term overall survival and cancer-specific survival [8, 9]. However, more than one-third of patients are ‘reclassified’ during follow-up, most of whom undergo curative treatment due to disease progression or patient preference [10]. These outcomes suggest high rates of misclassification which are likely reflective of the diagnostic tools available at the time [8, 11, 12]. Our study aims to determine the role of PSMA PET/CT in men with newly diagnosed low or favourable intermediate risk prostate cancer.

## Methods

This is a retrospective, single surgeon, single centre study performed at Sydney Adventist Hospital from January 2019 and March 2022. All men under the care of the single Urologist (H.W) who had low or favourable intermediate prostate cancer defined as: men with clinical stage T1-T2, PSA ≤15, Gleason score ≤3 + 4, less then 5% Gleason pattern 4, less then 50% of cores involved and absence of cribriform architecture or intraductal carcinoma on prostate biopsy (Table 1) [13] [14], underwent PSMA PET prior to placement on Active Surveillance. The total number of patients that met the inclusion criteria was 30 men (Additional File 1).

**Table 1:** Selection Criteria

Study Recommendation	Clinical stage	Histological characteristics	PSA
Royal Marsden Hospital	T1-T2	• Gleason ≤3 + 4. • ≤50% core involvement • ≤5% Gleason pattern 4 • Absence of cribriform architecture or intraductal carcinoma on prostate biopsy	PSA ≤15

The standard of care for men being evaluated for prostate cancer was to undergo a mpMRI scan prior to biopsy using a transperineal approach. The PRECISION Study method for prostate cancer diagnosis was incorporated, with targeted prostate biopsies being performed for men who had a positive result on mpMRI that is, in whom an area with a PIRADS score of 3 (equivocal regarding the likelihood of prostate cancer), 4 (likely to be prostate cancer), or 5 (highly likely to be prostate cancer) was identified. Systematic biopsies were taken in the context of a rising PSA, despite normal mpMRI results.

All PSMA PET scans were centrally read by an experienced dual trained nuclear medicine and radiology specialist (L.T).

Both 68Ga-HBEDD-11 and 18 F DCFPYL tracers were used during the defined study period. The scan protocol was an uptake time of 60 and 90 min respectively, scanning from the vertex to the thighs with a non-contrast-enhanced low dose CT scan post tracer injection. A diagnostic contrast CT chest, abdomen and pelvis was also performed as part of a usual standard of care examination. Intravenous contrast was administered at 1ml per kilogram. Standardised uptake value (SUVmax) was reported on a per lesion basis. In the absence of a globally accepted reporting system for PSMA PET CT thresholds for mild, moderate and marked/high levels of PSMA expression were defined by a combination of:

1. SUVmax ( < = 3–4 mild; 4–6 moderate, >=6–7 marked).
2. Tumour to background ratio (qualitative).
3. The reader’s level of diagnostic certainty (based on the MSKCC lexicon of certainty) using SUVmax and TB ratio, as well as mpMRI correlation with software PSMA PET MRI fusion in a large proportion of cases.

Primary outcome was to assess for change in management following PSMA PET/CT results.

The criteria for requiring further management was determined based on discordance between pathological and radiological findings. Men with PIRADS lesion 4 or 5 who had low or favourable intermediate prostate cancer on biopsy had further management if they also had moderate to marked levels of PSMA expression (as described above). For men with PIRADS 3 lesion further management was determined if they had either PSMA PET results including moderate and marked levels of PSMA expression or MRI occult lesions on PSMA PET.

Summary data is expressed in terms of medians with interquartile ranges. The study is approved (AHCL/HREC/2022-008) by the Adventist HealthCare Limited (AHCL) Human Research Ethics Committee (HREC).

## Results

**Fig. 1:**
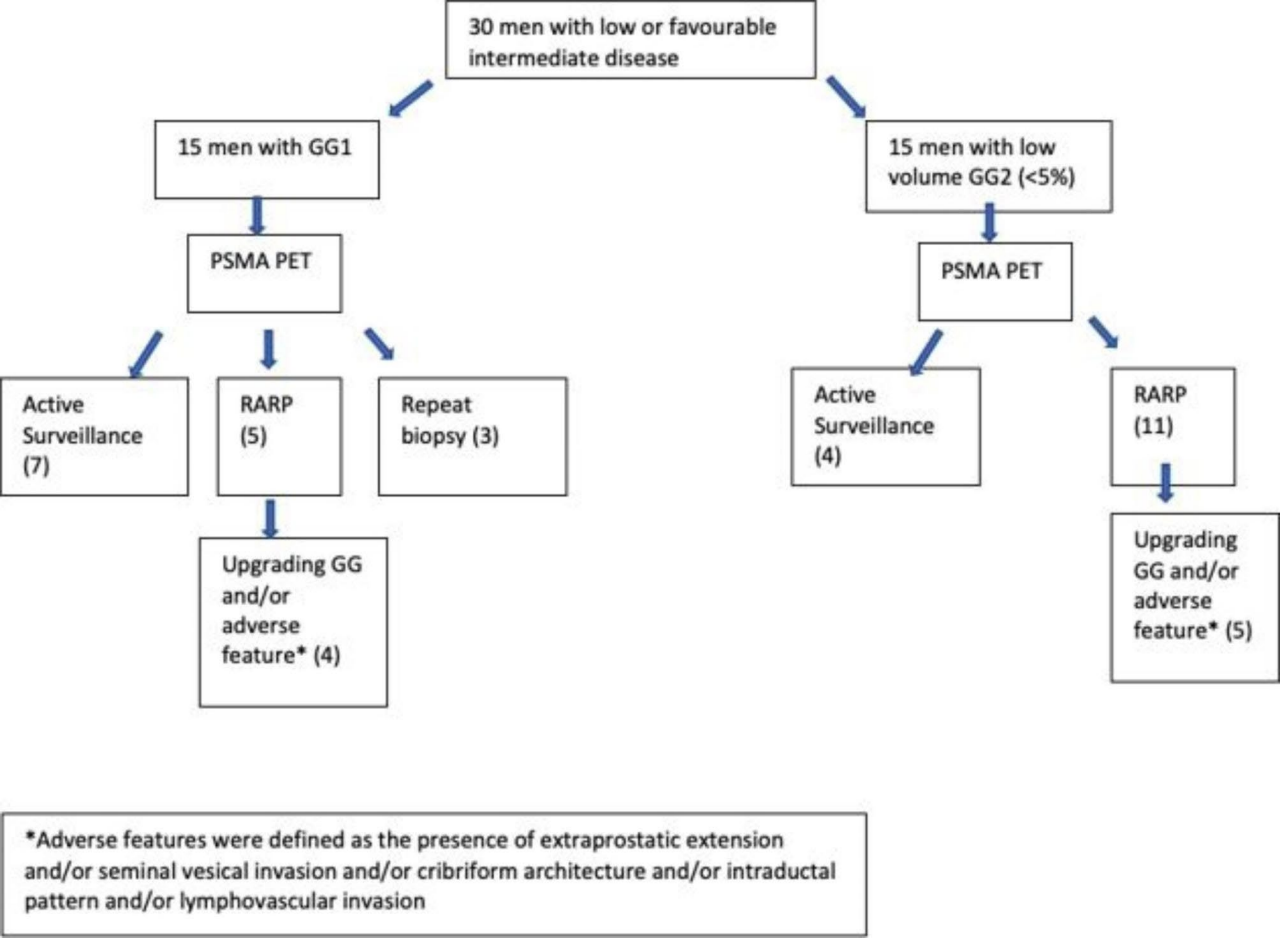
Summary of Management of Men with Low or Favourable Intermediate Risk Prostate Cancer

A total of 30 men were identified to have undergone a PSMA PET/CT scan following their diagnosis of low or favourable intermediate risk prostate cancer. Median age (IQR) and PSA was 64 (51.75–76.25) years and 5.5 (2.65–8.35) ng/ml respectively (Table 2). One (3.3%) man had PI-RADs ≤2, 4 (13.3%) men had PI-RADS 3, 12 (40%) PI-RADS 4 and 13 (43.3%) had PI-RADS 5 lesion. There were 15 men with either Grade Group (GG) 1 and GG2 disease on prostate biopsy (Table 3). Figure 1 provides a summary of the management of the 30 men who underwent PSMA PET/CT scans following their diagnosis of low or favourable intermediate risk prostate cancer.

**Table 2:** Patient Characteristics

Patient Characteristics	Overall n = 30
Age, years, median (IQR)	64 (56–68)
PSA level, ng/ml, median (IQR)	5.5 (3.7–6.5)
**MRI**	
PI-RADs, n (%) 1–2	1 (3.3)
3	4 (13.3)
4	12 (40)
5	13 (43.3)
**Prostate Biopsy**	
Positive cores	3 (1–4)
Total cores	10.5 (7–18)
Grade Group 1	15
Grade Group 2	15
**PSMA**	
SUV Max, median (IQR)	4.4 (3.25–7.71)
**Prostatectomy Outcomes**	**Overall** **N = 18**
Grade Group, n (%)	
1	4 (22.2)
2	12 (38.7)
3	2 (6.5)
EPE, n (%)	5 (16.1)
SVI, n (%)	0
LVI, n (%)	0
Cribiform, n (%)	5 (5.76)
Intraductal, n (%)	1
pN1, n (%)	1

**Table 3:** MRI and prostate biopsy results

MRI	GG1	GG2
PIRADS 1–2	0	1
3	2	2
4	5	7
5	8	5

There were 15 men with GG1 on initial diagnostic prostate biopsy of which 7 were assigned management by AS. All 7 men who were managed with AS, had mildly expressing lesions on PSMA PET with no evidence of EPE or SVI. Five men underwent a robotic assisted radical prostatectomy (RARP) and 3 men had a repeat prostate biopsy. Of the men, 7 men had concerning features on PSMA PET including [2] MRI occult lesion, [4] marked PSMA uptake and [1] concerning features on PSMA (EPE). One man proceeded to RARP due to patient preference and was found to have GG1 disease. Overall, 4 men that underwent RARP upgraded on final prostatectomy specimen of which 1 man also harboured adverse pathological features (presence of cribriform and ECE).

There were 15 men with GG2 on the initial diagnostic prostate biopsy of which 4 were assigned management by AS and 11 men had RARP from which 3 men underwent RARP due to patient preference. In the 3 men that underwent RARP due to patient preference, 2 had no upgrading or adverse features and one man had GG2 disease with cribiform pattern. In the 8 men that had concerning features on PSMA PET two had MRI occult lesion, 6 men had marked PSMA uptake. The corresponding prostatectomy in men that had abnormal PSMA findings was that 5 men had adverse pathological features (upgrading and/or cribiform and/or ECE and/or intraductal pattern). For the one man that upgraded to Grade Group 3 the corresponding PSMA results showed marked uptake (SUVmax 8.42) (Table 4).

**Table 4:** Pathological Outcomes of men who had PSMA altering scan

Patient	MRI	Biopsy	PSMA	Treatment	Final Histology
Patient 1	PI-RADS 3	GG1	MRI occult lesion SUV max 7.73	TP biopsy	GG1
Patient 2	PI-RADS 3	GG1	MRI occult lesion, SUV max 3.73	RARP	GG3, ECE, Cribiform, MRI occult lesion was index lesion
Patient 3	PI-RADS 4	GG1	Marked uptake, SUV max 8.1	RARP	GG2
Patient 4	PI-RADS 5	GG1	Marked uptake, SUV max 10.7	RARP	GG2
Patient 5	PI-RADS 5	GG1	Moderate uptake SUV max 5.4	TP	GG1
Patient 6	PI-RADS 5	GG1	Moderate uptake SUV max 5.3	TP	GG1
Patient 7	PI-RADS 3	GG2	Focal moderate uptake SUV max 5.98	RARP	GG2, ECE
Patient 8	PI-RADS 4	GG2	MRI occult lesion, marked uptake SUV max 8.05	RARP	GG2, cribiform, MRI occult lesion was index lesion
Patient 9	PI-RADS 4	GG2	Focal uptake, concerning for ECE, SUV max 5.6	RARP	GG2, ECE, cribiform,
Patient 10	PI-RADS 4	GG2	Marked uptake SUV max 8.06	RARP	GG2
Patient 11	PIRADS 5	GG2	Marked uptake SUV max 8.42, ECE	RARP	GG3, Cribiform, Intraductal
Patient 12	PI-RADS 5	GG2	Marked uptake SUV max 6.66	RARP	GG2
Patient 13	PI-RADS 5	GG2	Marked uptake SUV max 7.94 ECE	RARP	GG2, ECE
Patient 14	PI-RADS 5	GG2	MRI occult lesion, SUV max 7.71	RARP	GG2

Overall there were 4 men that had MRI occult lesions. One man had GG1 disease and underwent a repeat biopsy of the MRI occult lesion, which again demonstrated GG1. In the 3 men that had GG2 disease and underwent RARP, 2 men had corresponding MRI occult lesion and index lesion on final prostatectomy specimen.

In total, there were 11 of 30 men (36.67%) who were assigned management by AS and 19 of 30 men (63.33%) who had definitive treatment. Of the 11 patients that were placed on Active Surveillance, 10 men currently remain on Active Surveillance. One patient underwent treatment (robotic assisted radical prostatectomy). Final pathology of the prostatectomy specimen in this patient confirmed Grade Group 2 prostate cancer with less than 5% pattern 4. 15 of the 19 men that needed treatment had concerning features on PSMA PET/CT results. Of the 15 men with concerning features on PSMA PET, 9 (60%) men were found to have corresponding adverse pathological features on final prostatectomy features (upgrading and/or cribiform and/or ECE and/or intraductal pattern).

## Discussion

The key finding of our study is the demonstration that a significant proportion of men suitable for active surveillance had management diverted to intervention based on combined MRI and PSMA PET findings. From the 30 men who met the eligibility criteria for active surveillance, 15 (50%) men had concerning features on PSMA PET including moderate-marked (SUV max > 5) PSMA expression of the index lesion, MRI occult lesion or evidence of EPE. From the 16 men that underwent a robotic assisted radical prostatectomy, 10 (33.3%) men harboured at least one adverse pathological feature including the presence of cribriform architecture, intraductal pattern, extracapsular extension and/ or upgrade in grade group. Recent population-based studies support our findings, demonstrating upgrading rates ranging from 36.4 to 46% and 24-24.7% for men with low risk and favourable intermediate risk prostate cancer respectively [15]. Similarly, Mufarrij et al. reported 45.9-47.2% of cases were pathologically upgraded to a Gleason score ≥ 7 [16]. Furthermore, despite reassuring results on PSMA PET, 4 men opted to undergo definitive treatment.

Introduction of multiparametric MRI (mpMRI) and MRI targeted biopsies has transformed diagnosis and treatment of prostate cancer. Our study identified that for men who had GG ≥ 2 cancers either on biopsy or prostatectomy, 50% had lesions with a PI-RADS score of 4/5. Similarly a meta-analysis of 17 studies involving men with suspected or biopsy-proven PCa, the average PPVs for GG ≥ 2 cancers of lesions with a PI-RADS score of 3, 4 and 5 were 16% (7–27%), 59% (39–78%), and 85% (73–94%), respectively [17]. Furthermore, the PRECISION trial compared MRI targeted prostate biopsy with standard template guided biopsy reporting an increase in the detection rate of clinically significant disease from 26 to 38%, while reducing the detection of clinically insignificant disease from 22 to 9% [18]. Several guidelines reflect these findings and strongly recommend the use of mpMRI in the re-evaluation of men on Active Surveillance [19, 20].

Recently, prostate-specific membrane antigen (PSMA) PET/CT has been well-explored and successfully translated for the clinical diagnosis of PCa [3, 21]. PSMA expression is strongly correlated with Gleason score of the primary tumour [7]. Moreover, studies have evaluated the diagnostic value of using a combination of PSMA PET and prostate MRI to detect prostate cancer. A retrospective analysis of men with low to intermediate-risk PCa found that PSMA identified GG≥ 2 malignancies more frequently than GG 1 with sensitivity of 88% versus 18% [6]. This is further supported by Raveenthiran et al. that retrospectively analysed 1123 men and identified 92% of csPCa by combining mpMRI and (68)Ga-PSMA PET/CT [22]. These findings were confirmed in the prospective multicentre trial (PRIMARY trial)[23]. The PRIMARY trial confirmed 90% sensitivity of PSMA PET/CT for detecting csPCa. It also demonstrated the compelling advantage of PSMA in men with negative or equivocal MRI. On biopsy, 28% of men with PI-RADS 2 and 47% with PI-RADS 3 had csPCa, with 90% of these malignancies identified by PSMA [5].

There are several limitations to this study, including the small population size, single surgeon and retrospective single centre design. This highly selected patient population introduces selection bias that might overstate the extent to which management was altered based on PSMA PET/CT results. Furthermore, we acknowledge the limitation of PSMA PET/CT in the assessment of men with low or favourable intermediate prostate cancer. There are no established guidelines or standardised reporting tools to establish PSMA expression associated with the detection of clinically significant prostate cancer. It is also recognised that not all prostate cancers will express PSMA and could therefore result in a negative PSMA PET but given that this represents a small percentage of cases, it is unlikely to significantly alter the findings and conclusions of this study.

However, the majority of men who underwent a PSMA PET/CT had GG2 on their biopsy which represents a cautious approach for these patients when considering management by AS.

## Conclusion

The role of PSMA PET/CT in the diagnostic pathway for men with localised prostate cancer continues to emerge. Larger scale studies are needed to assess the role of PSMA PET in select men prior to being considered for active surveillance.

## Electronic supplementary material

Below is the link to the electronic supplementary material.

Additional File 1: Raw data of all patients included in the study

## Data Availability

Authors can confirm that all relevant data are included in the article and its supplementary information files.
